# Asymmetrical Pore Design of Sustainable Underwear for Improved Intimate Comfort

**DOI:** 10.1021/acsomega.6c03941

**Published:** 2026-09-21

**Authors:** Xiao Tian, Ying Li, Li Li

**Affiliations:** † School of Innovation and Creation Design, 47891 Shenzhen Polytechnic University, Liuxian Avenue, Shenzhen 518055, China; ‡ Division of Integrative Systems and Design, 26680 The Hong Kong University of Science and Technology, Clear Water Bay, Kowloon, Hong Kong 999077, China

## Abstract

The large amount of waste underwear generated has a considerable impact on the environment. Searching for more sustainable underwear materials and designs is important for effective air, thermal, and moisture management. However, sustainable cotton underwear is much less effective in moisture transport and in inhibiting the growth of bacteria and mites. Here, a hierarchically porous knitted fabric (HPKF) made from sustainable cotton and polylactic acid (PLA) fiber materials, constructed using an asymmetrical knitting pore design, is developed. The HPKF not only shows excellent moisture management compared to cotton fabric but also achieves a remarkable 27% increase in water evaporation rate. Furthermore, this design outperforms existing directional water transport fabrics by introducing antibacterial and antimite properties in its inner layer. The sustainable design and manufacturing processes align with the current textile industry infrastructure, offering enormous potential environmental benefits.

## Graphical abstract

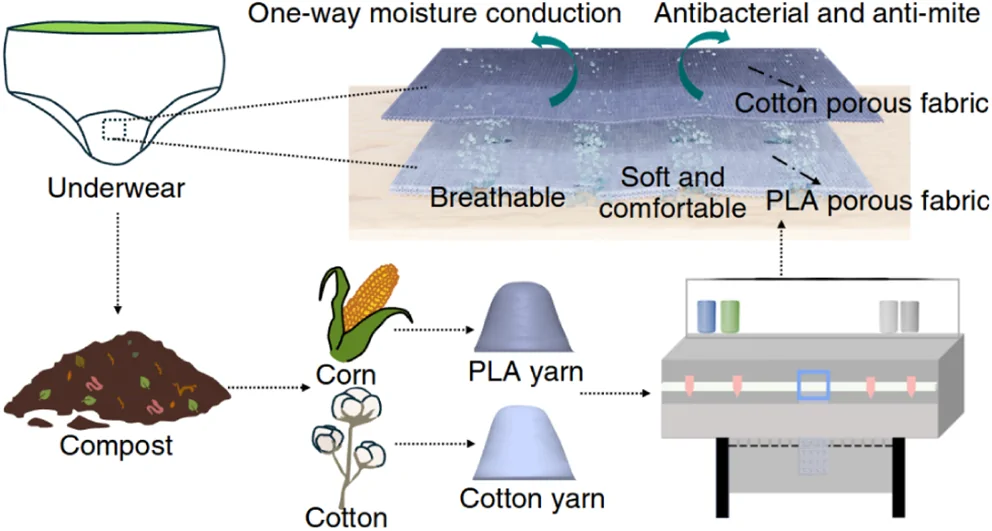

## Introduction

With the increasing awareness of health, it is crucial to change underwear frequently, as prolonged use of women’s underwear can harbor a significant amount of bacteria and harmful substances that pose health risks.^1,2^ However, a large quantity of discarded underwear may persist in the soil or enter water bodies for an extended period, causing adverse impacts on the ecosystem.^3–5^ Developing underwear with sustainable fiber materials is, therefore, critical for reducing the microplastics emitted during the life cycle of underwear. Currently, various fiber materials have been widely used in the development of underwear, including cotton,^6^ modal,^7,8^ silk,^9^ polyester, nylon,^10^ and spandex.^11^ Among these, cotton is the most commonly used material due to its natural and biodegradable properties.^12^ Nevertheless, cotton underwear lacks moisture and thermal regulation functions. For instance, although cotton fibers are breathable and have quick moisture absorption properties, they tend to retain humidity, releasing moisture slowly, which can lead to uncomfortable sensations of stickiness and excessive coldness.^13,14^ Prolonged dampness in cotton fabrics can create an environment conducive to the growth of bacteria and mites, thereby affecting health. In contrast, hydrophobic materials like polyester can effectively repel external water, but they are ineffective against internal sweat.^15^ Therefore, it is essential to explore a new underwear design with sustainable fiber materials that can maintain ideal moisture and thermal comfort.

Directional water transport (DWT) fabric, which transfers moisture rapidly away from the skin layer, has opened an important avenue.^16,17^ Developing DWT-based underwear effectively tackles the issues of sweat accumulation on the skin and stickiness resulting from limited sweat transport to the outer surface. Over the past few years, various DWT materials, such as nanofiber films,^18–21^ membranes,^13^ knitted fabrics,^22,23^ and woven fabrics,^24,25^ have demonstrated a superior directional water transport performance. However, the fabrication techniques, including electrospinning,^26,27^ screen printing,^28,29^ and laser processing,^30,31^ often involve complex processes and costly materials, which restrict their large-scale manufacture and subsequent market promotion. Additionally, most previous methods have relied on various toxic chemicals to alter the wettability of the fabrics. As a result, several important considerations must be addressed in the development of DWT-based underwear. First, ideal underwear needs to be antibacterial and antimite because it is in direct contact with sensitive areas. Good water vapor permeability and air permeability are desirable to avoid excessive dampness in these areas. Furthermore, sustainable textile materials and production processes are essential to ensure green and low-carbon development of the textile industry.

Herein, we present a hierarchically porous knitted fabric (HPKF) based on an asymmetrical knitting pore design and sustainable cotton and polylactic acid (PLA) fiber material configuration for directional water transport of the underwear. Specifically, the design of functional pores in the inner layer of the underwear realizes desirable air permeability of the bilayer, which is nearly equivalent to that of a single-layer PLA fabric. As a synergistic effect, the hierarchical porous structure of the bilayer enhances the water evaporation of cotton fabric and thermal conductivity of PLA fabric while providing protection against external microbial intrusion. Moreover, the design and fabrication processes are compatible with existing infrastructures of the textile industry, simplifying the transition to industrial manufacturing. Therefore, this work demonstrates a sustainable and scalable material and design strategy for the underwear that can improve the intimate care in terms of air, thermal, moisture transfer, and protection against external microbial invasion as well as offer enormous potential environmental benefits.

## Experimental Section

### Materials and Fabrication of Porous Fabrics

Polylactic acid filament (150D) was supplied by HI-TECH Fiber Group Corporation. 32S bleached cotton yarn was purchased from ESQUEL ENTERPRISES Co., Ltd. The porous cotton and PLA fabrics were fabricated using a flat knitting machine (ADF 830-24 W) with a gauge of 18 (needles/inch). The fabrics were designated on the basis of their fiber type and pore spacing. Specifically, C0 and P0 fabrics were designed with a single jersey structure. The remaining fabrics, C10, C5, C3, P10, P5, and P3, share a pointelle structure, where the number following the letter indicates the center-to-center distance between adjacent pores, with the corresponding pore spacings of 10 mm, 5 mm, and 3 mm, respectively. The cotton and PLA yarns were threaded into the machine, and appropriate knitting pattern files were uploaded. The carriage traversed the needle bed back and forth to create the fabric.

### Characterization

All fabric specimens were subjected to two consecutive washing and subsequent drying procedures according to AATCC 135 standard. These processes were aimed at effectively eliminating the water-soluble impurities and reducing the dimensional instability of the fabrics. A Leica M165C (DFC 290HD, Leica Microsystems Ltd.) was used to observe the surface morphologies of the knitted fabrics. The directional water transport capabilities of the fabrics were evaluated using the SDL Atlas Moisture Management Tester (MMT). To precisely assess the air permeability of the fabrics, an air permeability tester (KES-F8, Kato Tech Co., Ltd.) was utilized. The performance metric evaluated was airflow resistance (R), where increased resistance signifies reduced permeability. The KES-F7 Thermo Labo II Instrument was used to measure the fabric’s thermal conductivity. Water vapor transmission was examined using GB 12704.1 with a constant-temperature and -humidity chamber (38°C, 90% humidity). The water absorption rate and water evaporation were evaluated according to standard GB/T 21655.1. The water contact angles of the fabrics were examined using an optical CA measuring device (SDC-350, Sindin, China). A 5 μL water droplet was placed on the fabric surface, and the contact angles were measured and recorded. Each fabric sample was tested in triplicate, and images were captured every 0.3 s. The vertical wicking heights of the PLA and cotton layers were investigated following standard FZ/T 01071. Fabrics measuring 30 mm × 250 mm were prepared. The fabric was hung vertically, with its lower edge immersed in water to a depth of 15 mm for 30 min. After this period, the capillary rise of the water in each sample was measured to evaluate the fabric’s wicking ability. An infrared camera (FLIR E33) was employed to capture thermal images.

## Results and Discussion

The designed HPKF mainly comprises two components: cotton and PLA fibrous layers with asymmetrical pores. Figure 1a shows the schematic illustration of the process from raw material to yarn, fabric, and finally to the waste generated by the underwear. The inner layer, in contact with the private parts, is made of PLA fibers derived from starchy agricultural products, such as corn, wheat, and sugar beets. This layer features a lower moisture regain and faster moisture transport, along with good biodegradability and antibacterial properties. Meanwhile, cotton fibers from cotton plants are selected for the outer layer because of their excellent water absorption, natural breathability, skin comfort, and biodegradability. The flat knitting technique is utilized to create fabric with multiscale pores using PLA and cotton yarns (Figure 1b) owing to its high production efficiency, versatile design options, and seamless characteristics (Figure S1a). Figure S1b shows the knit chart of porous fabric. The manufacturing process of these porous fabrics is illustrated in Video S1. The resulting HPKF presents a bottom-up multiscale network with both basic and functional pores (Figure 1c). The combination of PLA and cotton fibers provides a water absorption gradient through the thickness, leading to an asymmetric wetting profile in HPKF. Such a porous fibrous bilayer can be applied to the bottom of underwear. Ultimately, after a period of use, underwear is discarded. The PLA and cotton materials in discarded underwear can be easily separated. The cotton fibers can naturally decompose, while PLA can undergo composting degradation, breaking down into lactic acid, which further hydrolyzes into carbon dioxide and water.^32–34^ Through photosynthesis, plants can convert carbon dioxide and water back into starch, creating a natural cycle that contributes to ecological balance. Figure 1d,e display the optical micrographs of cotton and PLA yarns, respectively. In Figure 1f,g, it can be observed that the PLA fabric consists of multiscale pores constructed by a single jersey structure and pointelle, while the cotton fabric includes one type of pore formed by a single jersey structure.

**Figure 1:**
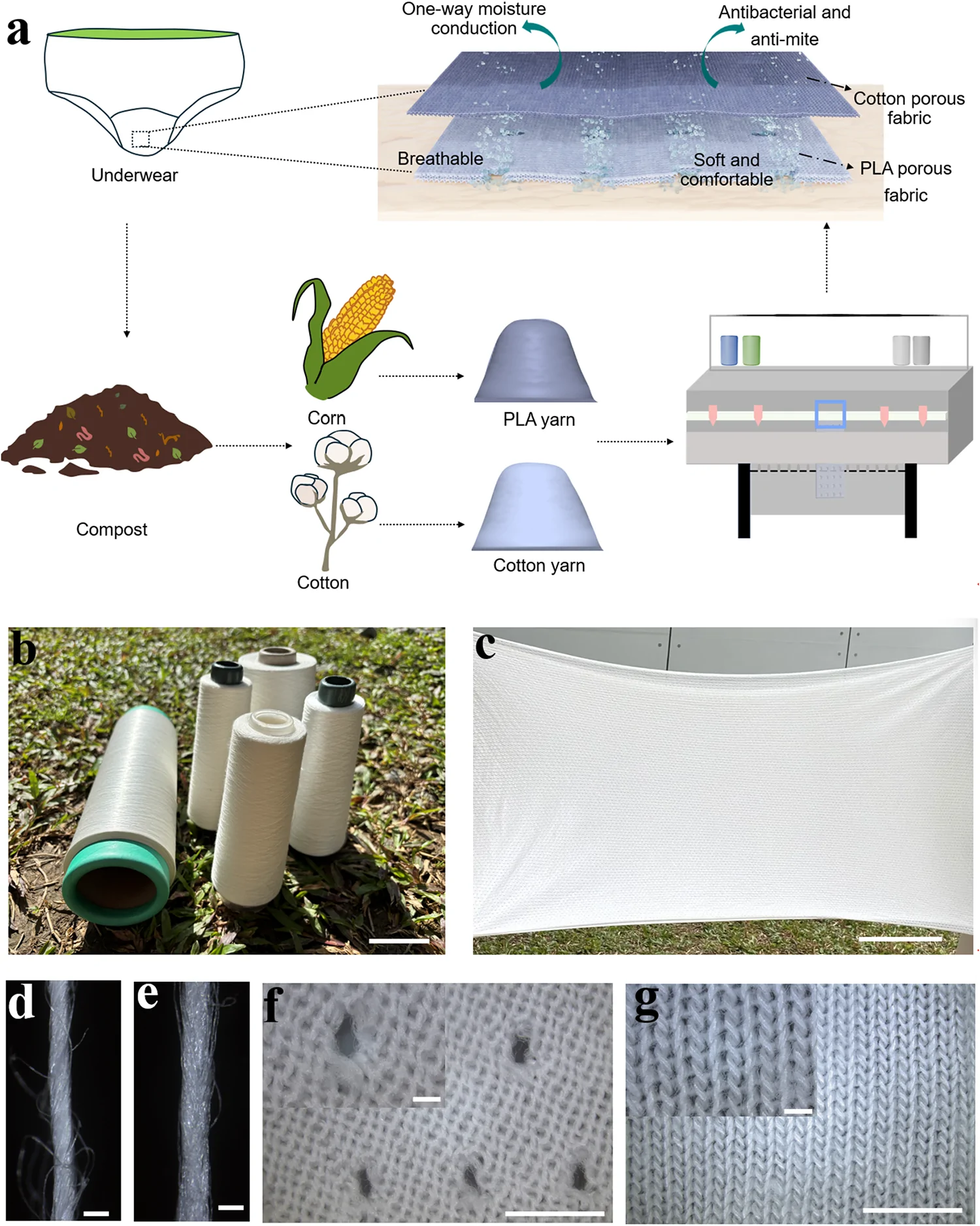
(a) Life cycle diagram of the fabricated underwear from raw materials through production to finished products. (b) Photograph of the PLA and cotton yarns. Scale bars: 4 cm. (c) Photograph of the HPKF (1.5 m × 0.75 m). Scale bars: 25 cm. Optical micrographs of (d) cotton yarn and (e) PLA yarn. Optical images of the (f) PLA and (g) cotton layer of the HPKF. Scale bars: 1 mm and 5 mm.

### Water Transport Behavior in the HPKF

To systematically study the effects of the layer system and functional pore configuration on water transport behavior, single- and double-layer fabrics with no functional pore, as well as functional pore distances of 10 mm, 5 mm, and 3 mm, were designed and prepared using cotton and PLA fibers. The single-layer cotton fabric and PLA fabric are defined as follows: no functional pore (C0) and functional pore distances of 10 mm (C10), 5 mm (C5), and 3 mm (C3). Similarly, the single-layer PLA fabrics are designated as P0, P10, P5, and P3, corresponding to the same pore distances. The specifications of the prepared fabrics are presented in Table S1. The weight and thickness of the fabrics are shown in Figure S2. It can be observed that as the number of functional pores increases, the weight of the PLA porous fabric decreases, while its thickness increases. This is due to the small protruding morphology of the functional pores created by the knitting of the PLA yarns. However, for the cotton porous fabric, the change in the weight is not significant. Nevertheless, the addition of pores does result in an increase in the thickness of the cotton fabric. The photographs and surface morphologies of these fabrics are presented in Figures S3 and S4.

In terms of layer systems, cotton double layers, PLA double layers, and combinations of PLA and cotton double layers are also explored. The cotton double layers are defined as C0C10, C0C5, C0C3, and C0C0, where C0 serves as the outer layer and C10, C5, C3, and C0 function as the inner layers. The PLA double layers are designated as P0P10, P0P5, P0P3, and P0P0, with P0 as the outer layer and P10, P5, P3, and P0 as the inner layers. Additionally, the combination of PLA and cotton double layers, which uses C0 as the outer layer and P10, P5, and P3 as the inner layers, is named C0P10, C0P5, and C0P3. For comparison, the combinations using P0 as the outer layer and C10, C5, and C3 as the inner layers are named P0C10, P0C5, and P0C3. The water transport ability of the fabrics was characterized using a moisture management tester (MMT), as shown in Figure S5. The one-way transport index (R) and overall moisture management capacity (OMMC) were used as indicators to evaluate the one-way water transportation performance of the fabric. The index R represents the difference in cumulative moisture content between the two surfaces of the fabric during the testing period, serving as a direct indicator of the fabric’s ability to absorb moisture and move internal water outward. The R values for the P0C10, P0C5, and P0C3 fabrics, moving from the cotton side to the PLA side, are −174%, −148%, and −213% (Figure 2a), respectively, indicating poor one-way water transfer behavior. In contrast, when comparing the R values of single-layer cotton fabrics with varying pore distances, single-layer PLA fabrics, and double fabrics of both materials, the C0P5 fibrous double layer transitioning from PLA to cotton demonstrates exceptional forward transport capability with an R value of 322%. This performance is attributed to the gradient difference in water absorption between PLA and cotton, as well as the capillary forces within the pores. The water location in these fabrics is indicated in Figures S6–S11. OMMC refers to the weighted sum of the water absorption rate on the lower surface of the fabric, the one-way transmission index, and the diffusion rate on the lower surface. The overall liquid water dynamic transmission index provides a comprehensive measure of liquid water movement across the fabric. As shown in Figure 2b, C0P5 exhibits the highest OMMC at approximately 0.69, which can be attributed to its material configuration and structural design. When the red liquid droplet comes into contact with the inner layer, a downward hydrostatic pressure is immediately generated, which is influenced by the droplet size. Owing to the strong capillary action of the PLA yarns, the liquid water in the droplet is rapidly absorbed and begins to spread in the horizontal direction. Meanwhile, the favorable moisture-wicking properties of the PLA yarns, combined with the fabric’s structural design, enable the moisture to be transported to the outer cotton yarns under the combined driving forces of capillary pressure differences and hydrostatic pressure, where it is primarily absorbed by the cotton yarns and subsequently evaporated. This yarn configuration facilitates the rapid expulsion of human sweat, thereby maintaining skin dryness and comfort. In terms of R and OMMC, the C0P5 exhibits the best performance. This is mainly attributable to the fact that, with C0 as the outer layer, the pore spacing of the inner P5 layer achieves an optimal balance between capillary driving force and channel density. In contrast, the pore spacing of P3 is too dense, giving rise to competitive capillary effects among the pores that interfere with continuous liquid transport, whereas the spacing of P10 is too sparse, resulting in insufficient driving force and reduced transport continuity. Therefore, C0P5 is recognized as the optimal combination for water transportation capacity, identified as the HPKF.

**Figure 2:**
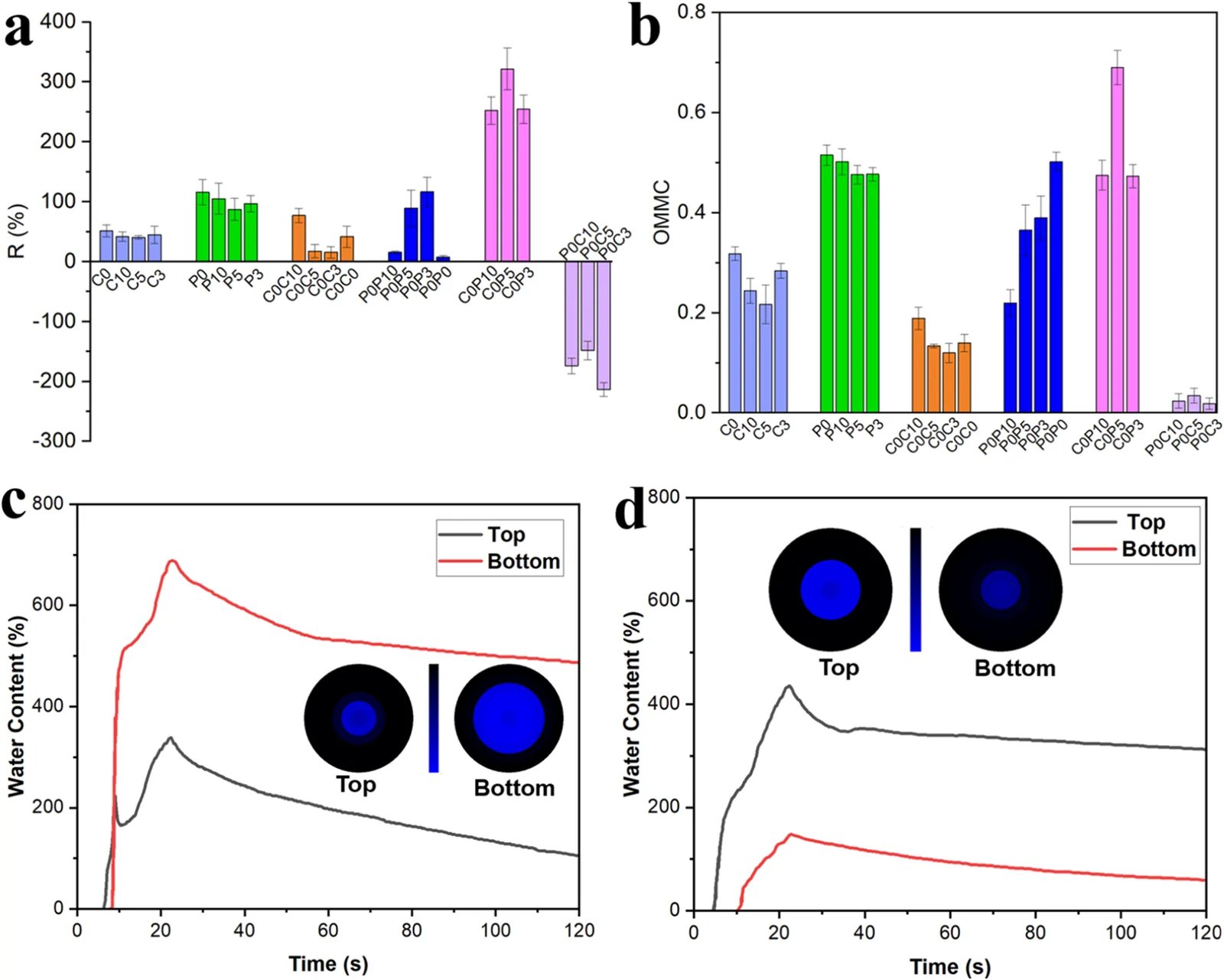
Comparison of (a) one-way transport capability and (b) OMMC of the fabrics with different materials, pores, and layers. MMT results of the HPKF when the water was supplied on the (c) PLA fabric and (d) cotton fabric.

Figure 2c,d illustrates the water content variations of the cotton and PLA fabrics of the HPKF. When the PLA fiber layer is facing up, the moisture content on the PLA surface peaks at 338% after 22 seconds, subsequently decreasing to 105% at 120 s once the saline water supply stops. Because of the design of the moisture-wicking channels in the fabric, water is rapidly drawn to the surface of the cotton layer, which reaches a maximum moisture content of 689% at 22 seconds. In contrast, when the cotton fiber layer is facing up, the moisture content on the surface peaks at 436% after 22 seconds and then decreases to 312% at 120 s. Meanwhile, the moisture content of the PLA fiber layer also peaks at 148% after 22 s, declining to 59% at 120 seconds. This further illustrates that the hierarchical porous fibrous bilayer exhibits an excellent moisture management property.

To further observe and understand the water transport process of the HPKF, a red ink droplet of 200 μL was dropped on the PLA and cotton layers, respectively. When a water droplet was added onto the PLA layer (Figure 3a), it initially retained a nearly hemispherical form. Subsequently, the water droplet infiltrated and dispersed across the cotton fibrous side of the hierarchical porous fibrous bilayer, resulting in a small ink mark on the upper surface. In contrast, the water droplet placed on the cotton fibrous side rapidly spread along the surface (Figure 3b), while the PLA fabric retained a small ink droplet and appeared almost dry. The cotton layer exhibited a significantly larger spreading area than the PLA layer due to the pronounced hydrophilic behavior of cotton fibers and the gradient pore size of capillary channels in the hierarchical porous fibrous bilayer. Furthermore, the variation of the water contact angle on the PLA and cotton layers was evaluated, as shown in Figure 3c. The water contact angle of the PLA layer is maintained at about 115° for 0.9 s. After that, the water absorption rate becomes faster, and the droplets are completely absorbed in 1.5s. In contrast, the water contact angle in the cotton layer remains approximately at 0°, demonstrating the excellent hydrophilicity of the cotton layer. As a result, water droplets penetrate from the PLA layer into the cotton layer, where they subsequently evaporate. This phenomenon is attributed to the moisture absorption properties of both PLA and cotton, as well as the configuration of their structural pores. The water transport performance of the PLA and cotton layers was also characterized by wicking height and speed. As shown in Figure 3d, the fabric exhibits a faster wicking rate in the initial stage, attributed to the synergistic effects of the yarn’s inherent hydrophilicity and the capillary action generated by the pores within the fabric. Additionally, the wicking height of the cotton layer is higher than that of the PLA layer due to its superior water absorption capacity.

**Figure 3:**
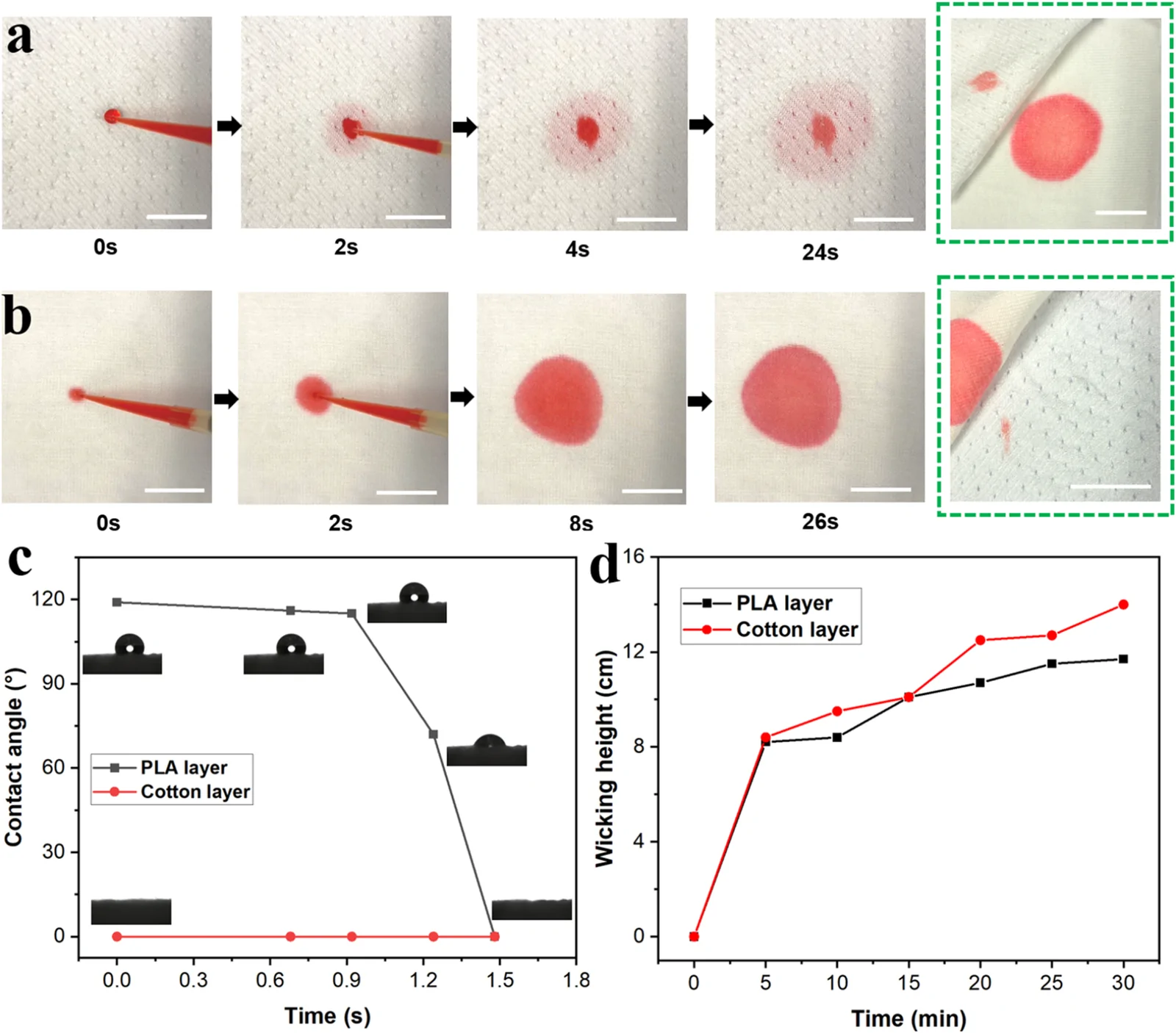
Water transport process of the HPKF when the red ink droplet is dropped on the (a) PLA and (b) cotton fabrics. Scale bars: 2 cm. (c) Variation of the water contact angles of the PLA and cotton layers. (d) Wicking heights of the PLA and cotton layers.

### Comfort Properties of the HPKF

Evaluating fabric’s thermal conductivity is crucial for assessing its ability to regulate temperature and moisture, which directly influences comfort performance and suitability for specific applications in clothing and textiles. The schematic of the thermal conductivity testing equipment is shown in Figure 4a. Initially, room-temperature water was circulated in the “water box” set to 25 °C, while the temperature of the BT-Box was adjusted to 35°C. The sample was placed on the “water box”, and after both the “BT-Box” and the guard reached their designated temperatures, the “BT-Box” was positioned on the sample to ensure full contact. The “W” button was pressed, and the stabilized reading was recorded. The weight of the “BT-Box” was 150 g, resulting in a pressure of 6 gf/cm^2^ during measurement. This procedure was repeated five times. The thermal conductivity of the sample is determined using the following equation

1 $$k=\frac{W\times D}{A\times \Delta T_{0}}$$

where *W* represents the heat consumption in watts. *D* signifies the sample thickness in cm, and *A* denotes the area of the BT-Box in cm^2^. Additionally, Δ*T*_0_ refers to the temperature difference between the BT-box and water box in °C. The area *A* was 25 cm^2^, and Δ*T*_0_ was 10 °C.

**Figure 4:**
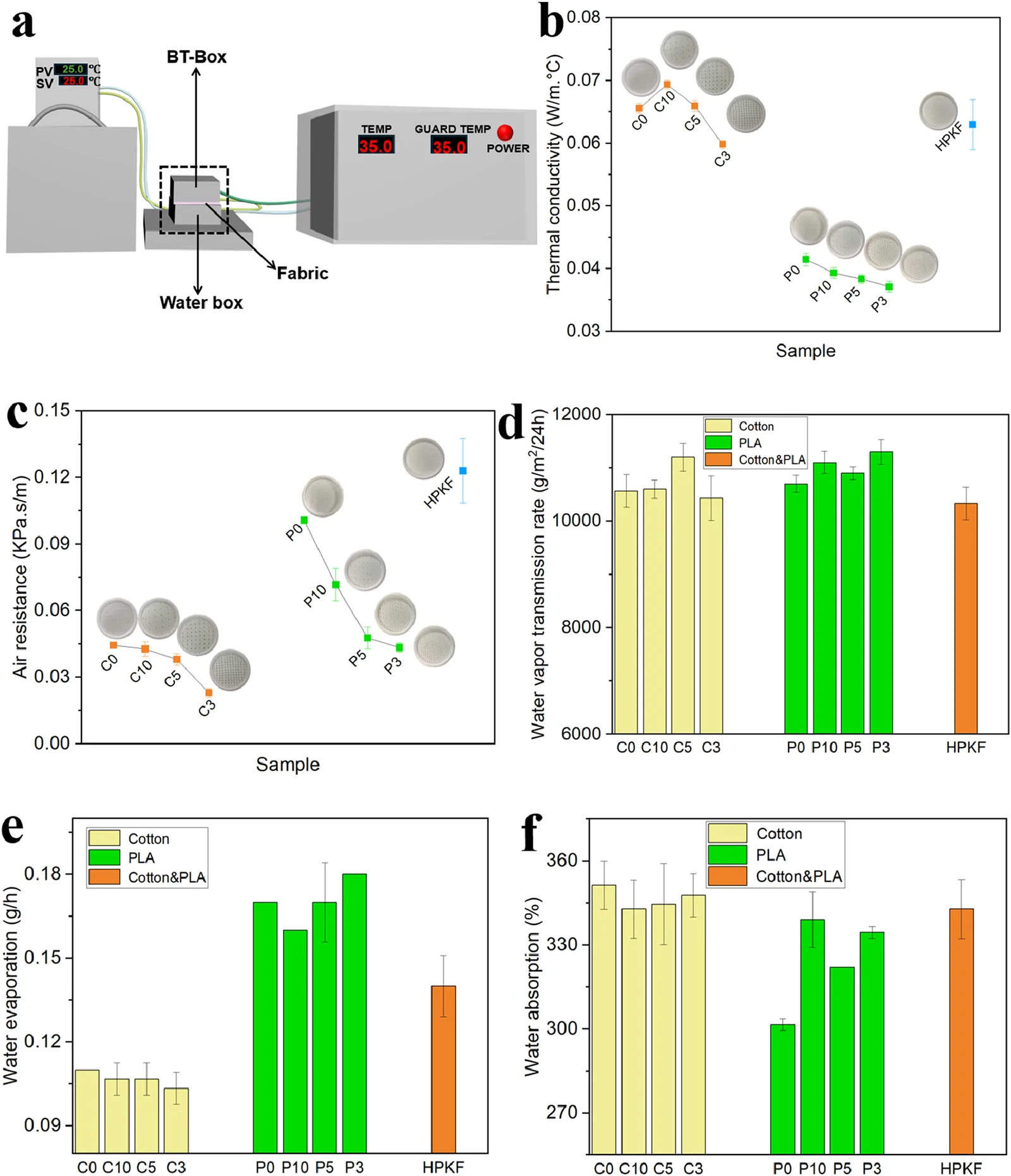
(a) Schematic of thermal conductivity testing equipment. (b) Thermal conductivity and (c) air resistance of the HPKF. (d) Water vapor transmission, (e) water evaporation, and (f) water absorption of the HPKF. For data points without visible error bars, the calculated error value equals zero.

Figure 4b shows the results of the thermal conductivity of HPKF. The thermal conductivity decreases as the pore spacing of the cotton and PLA single layers reduces from 10 to 3 mm. The thermal conductivity of air is approximately 0.025 W/mK, while that of cotton is typically in the range of 0.03–0.05 W/mK. Both cotton and PLA exhibit higher thermal conductivity than air. The porosities of P10 and C10 are lower than those of P3 and C3, indicating a reduction in pore spacing and an increase in porosity. The higher porosities of P3 and C3 imply a lower fiber volume fraction and a higher air content within the fabric, which results in lower thermal conductivity. Additionally, the HPKF demonstrates good thermal conductivity comparable to that of the C3 and C5 fabrics. It is evident that cotton fabric has a higher thermal conductivity than the PLA fabric. In textile materials, the presence of air within the fabric structure significantly impacts the thermal conductivity values. While air possesses the lowest thermal conductivity (approximately 0.025 W/mK) among all fibers, water has the highest thermal conductivity (around 0.6 W/mK).^35^ Since PLA fiber has a moisture regain of only 0.4%, PLA fabric demonstrates poor thermal conductivity compared to cotton fabric. In contrast, cotton fabrics can release trapped moisture from the fiber matrix when they are heated, which can carry a considerable amount of heat energy. This process enhances heat transfer across the fiber layer, resulting in improved thermal conductivity. The air resistances of the cotton and PLA fabrics were also measured. As the pore distance of the fabric decreases, its air resistance also diminishes (Figure 4c). This is because fabrics with smaller pore distances tend to have a higher porosity. The HPKF has excellent air permeability (0.123 KPa s/m) comparable to that of the single-layer PLA fabric (0.1 KPa s/m).

The water vapor transmission rate (WVTR), water absorption, and water evaporation of the fabrics were subsequently measured. The WVTR measures how effectively a fabric allows insensible perspiration to escape, which is crucial for enhancing comfort. The HPKF exhibits an excellent WVTR (10,333 g/m^2^/24 h), comparable to that of the single-layer cotton fabric (10,433 g/m^2^/24 h) (Figure 4d). This advantage stems from the spaces between the fibers and the optimal pore channel network. Additionally, the fabric with high water absorption capacity can efficiently absorb sweat from the body, helping regulate temperature and improve comfort. Moreover, a higher rate of water evaporation is essential for facilitating sweat transport and evaporation from the skin, contributing to the fabric’s cooling performance. In evaporation tests, the HPKF demonstrates an evaporation rate approximately 27% faster than that of single-layer cotton (Figure 4e). The water absorption of the HPKF exceeds that of single-layer PLA and is nearly equivalent to that of single-layer cotton (Figure 4f). Notably, the double-layer configuration does not adversely affect air, thermal, or moisture comfort.

### Antibacterial and Antimite Properties of the Inner Layer

The antibacterial and antimite properties of underwear are essential for preventing infections, reducing allergies, enhancing comfort, and maintaining overall health. The oscillation method was selected for antibacterial testing of the fabric following the standard GB/T 20944.3-2008. For this investigation, Gram-positive *S. aureus*, Gram-negative *E. coli*, and *C. albicans* were chosen to evaluate the biocidal efficacy of the inner layer of the HPKF. Cotton fabric was used as a control blank sample. The inner layer P5 and the blank sample were cut into 5 mm × 5 mm pieces. A total of 0.75 g of the P5 sample and 0.75 g of the blank sample were weighed out. Subsequently, 5 mL of diluted inoculum was added to a 250 mL conical flask containing 0.75 g of the P5 sample and 70 mL of PBS buffer, which had been sterilized by autoclaving. The test samples were incubated in a shaking incubator at a temperature of 24 ± 1 °C and a speed of 250–300 rpm for 18 h, with the blank sample serving as the control. After incubation, the solutions were diluted to an appropriate concentration. A volume of 1 mL ± 0.1 mL of the diluted test solution was transferred to a sterile Petri dish, and 15 mL of agar medium was added. The agar plate was then incubated, and the number of surviving bacteria was recorded. The inhibition rate is determined using eq 2

2 $$R=\frac{X-Y}{X}\times 100\%$$

where *R* represents the inhibition rate of the sample, *X* is the number of survival bacteria in the blank sample, and *Y* is the number of survival bacteria in the tested sample.

Table 1 presents the number of bacteria and the inhibition rate of the P5 and blank samples. The results illustrate that the antibacterial rates of the P5 layer against *S. aureus*, *E. coli*, and *C. albicans* are 99%, 98%, and 93%, respectively, exceeding the standard requirements of 70%, 70%, and 60%. This indicates that the antibacterial efficacy of P5 is significant. The observed effectiveness is likely attributed to lactic acid, a potent organic acid known for its antibacterial and antifungal properties.^36,37^ Small quantities of lactic acid are retained and migrated from the PLA fiber matrix, which likely contributes to the observed bacteriostatic effect.

**Table 1:** Antibacterial Effectiveness Following the Inoculation of *S. aureus*, *E. coli*, and *C. albicans* Suspensions Incubated with the Inner Layer P5 for 18 h

	*S. aureus*		*E. coli*		*C. albicans*	
bacteria	blank (CV%)	P5 (inner layer)(CV%)	blank (CV%)	P5 (inner layer)(CV%)	blank (CV%)	P5 (inner layer)(CV%)
The number of bacteria at 0 h contact time (CFU ml^–1^)	2.1 × 10^4^(0)		2.2 × 10^4^(0)		1.9 × 10^4^(0)	
The number of bacteria at 18 h contact time (CFU ml^–1^)	2.9 × 10^6^ (2.0)	5.2 × 10^3^ (4.8)	8.2 × 10^6^ (2.4)	2.0 × 10^5^ (2.9)	6.5 × 10^5^ (5.5)	4.5 × 10^4^ (5.6)
Inhibition (%)	>99		98		93	

The antimite properties of underwear are crucial for effectively reducing allergic reactions and skin issues caused by dust mites, thereby protecting the wearer’s health. The effectiveness against dust mites was evaluated using the repellency method of GB/T 24253-2009, “Evaluation of Anti-Mite Performance of Textiles.” Three control samples and three test samples, each weighing a total of (0.4 ± 0.05) g, were placed under drying conditions at (65 ± 5) °C for 10 min. A saline solution was added to a container (200 mm × 200 mm × 10 mm) with a sponge until the sponge was fully submerged. Figure 5a shows the schematic of the repellency method for testing antimite properties. The test and control samples were placed in outer Petri dishes containing 0.05 g of mite powder arranged in a petal pattern. Approximately (2000 ± 200) mites were subsequently added to the central Petri dish. The dishes were sealed and incubated for 24 h at (25 ± 2) °C and a relative humidity of (75 ± 5)%. To evaluate the mite repellent effectiveness, the number of live mites in the test and control dishes was recorded. The number of mites in the control samples was 214, 168, and 221, respectively, while the number of mites in the test samples was 48, 52, and 53 (Figure 5b). The mite repellent rate *Q* was calculated according to eq 3

3 $$Q=\frac{B-T}{B}\times 100\%$$

where *B* represents the average count of live mites in the three control samples and *T* indicates the average count of live mites in the three test samples.

**Figure 5:**
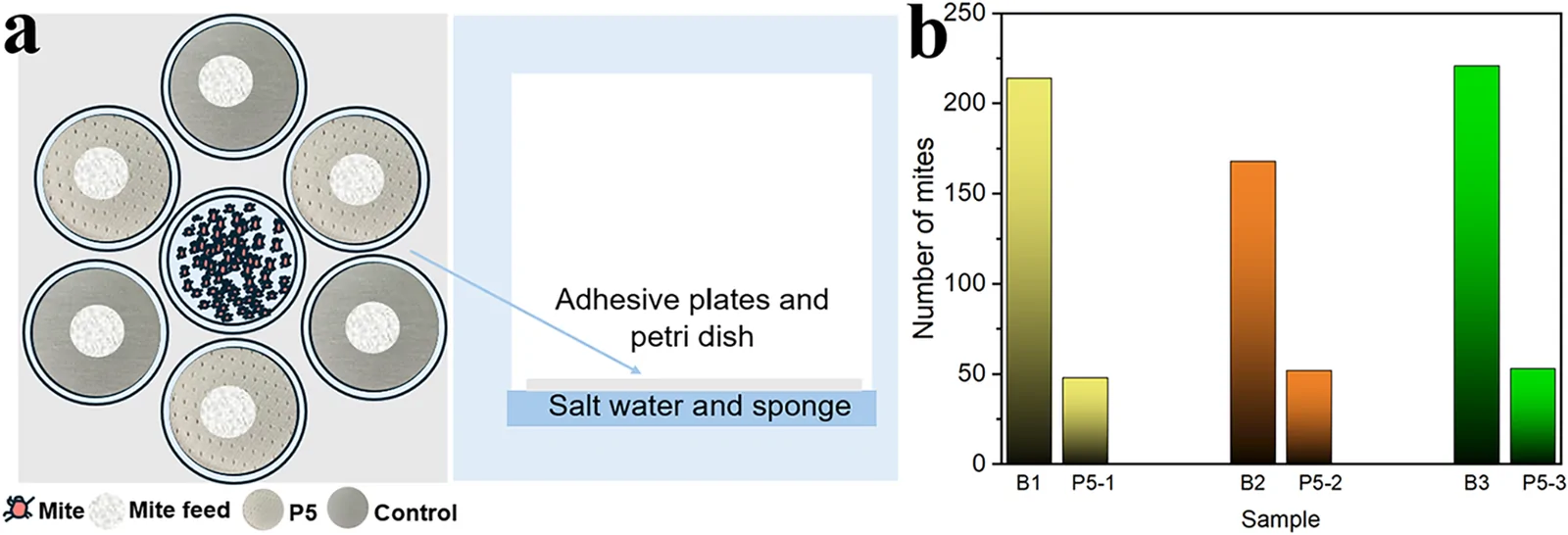
(a) Schematic of the repellency method for testing antimite properties. (b) Antimite property of the inner layer.

The antimite rate of the inner layer P5 can reach 75%, demonstrating a significant antimite effect. This effectiveness can be attributed to the low moisture absorption of PLA fibers, which makes the fabric less likely to absorb water in humid environments. As a result, the habitat for dust mites is reduced compared with traditional cotton fabrics, contributing to improved health and comfort for the wearer.

### Application of the HPKF

To illustrate the superior performance of the developed HPKF, the underwear was fabricated using the elastic band, developed cotton fabric, and HPKF, as shown in Figure 6a. Various commercially available underwear made from different materials and structures were selected for comparison, including cotton, viscose fibers, modal, silk, spandex, nylon, and blended cotton/PLA yarns. They are named as 1#, 2#, 3#,4#, 5#, 6#,7#, 8#, and 9#. The detailed materials content is presented in Table S2. A 200 μL red ink droplet was placed on the inner layer of the developed HPKF and commercial underwear, respectively. The photographs were obtained after 120s. The results indicate that the inner layers of 1#, 3#, 5#, 6#,7#, 8#, and 9# exhibit a higher diffusion area compared to that of the HPKF, as shown in Figure 6b. In contrast, the red droplets on #2 and #4 are difficult to move down within 120 seconds (Figure 6c). The results indicate that our underwear exhibits excellent moisture management.

**Figure 6:**
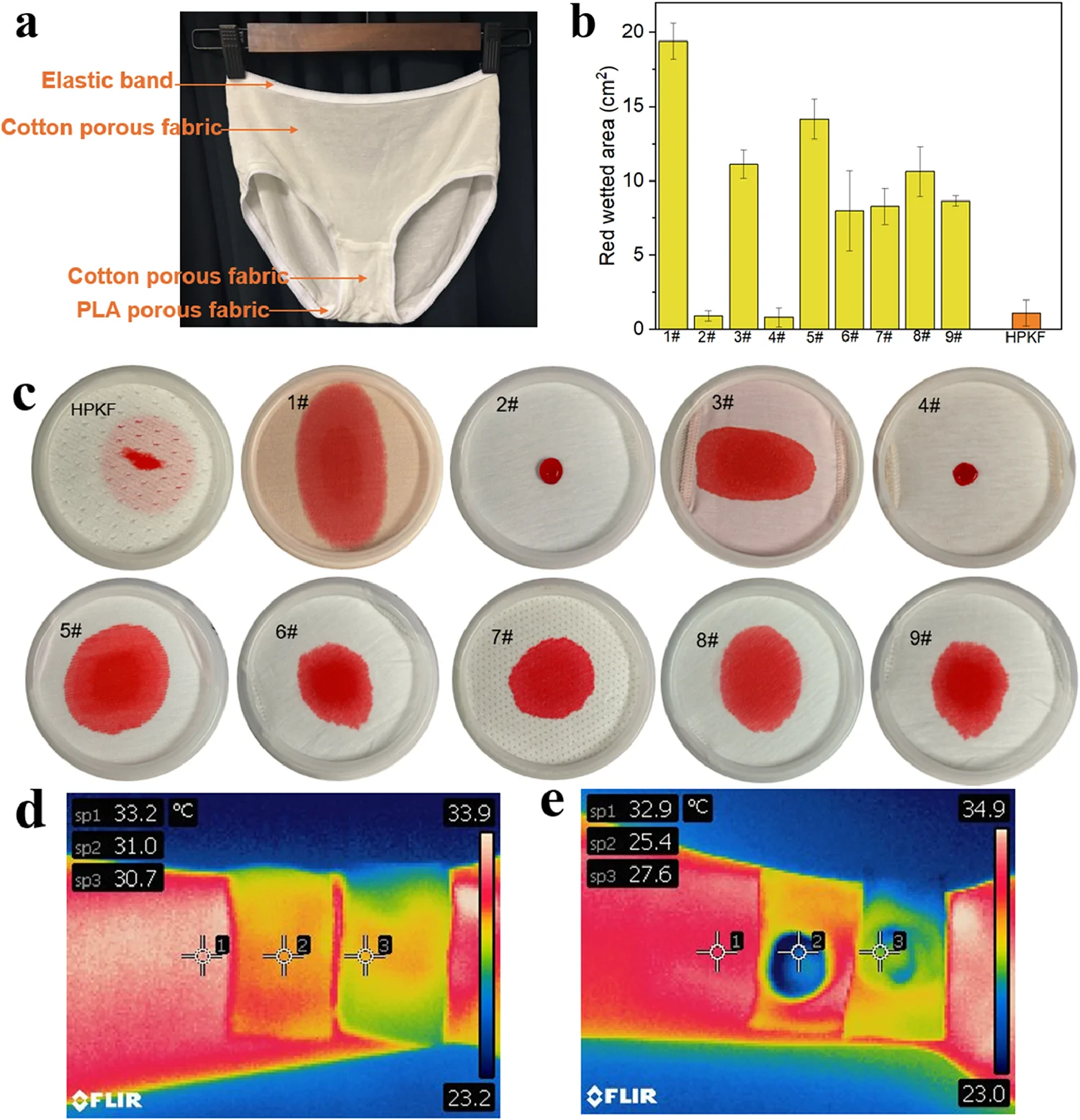
(a) Photograph of the underwear. (b) Red droplet wet diffused area. (c) Photographs of both HPKF-developed underwear and commercial underwear when red liquid drips onto their inner layers. Infrared camera images of the HPKF and commercial cotton textile placed on (d) dry skin and (e) wet skin.

An infrared camera was used to test the temperatures of the HPKF and a commercial cotton double-layer bottom on the dry skin surface. First, the HPKF and a commercial cotton double-layer bottom with a similar size (approximately 3 cm × 4.5 cm) were attached to the skin of the dry arm, ensuring they were securely adhered. The samples were gently pressed onto the skin. After 1 min, an infrared thermal imaging camera was employed to simultaneously measure the temperatures of both samples and the arm. As shown in Figure 6d, the temperature of the HPKF is approximately the same as that of the cotton fabric, at 31 °C. The HPKF does not have a significant cooling effect when the body is in a dry state. Subsequently, two drops of water were applied to the surface of the arm. Following this, two samples were positioned on the arm where the water was applied, and they were gently pressed until fully saturated. After another minute, a thermal imaging camera was used to measure the temperatures of both wet samples. As shown in Figure 6e, the HPKF is in a cooler state with a low temperature of 25.4 °C (Sp2), which is 2.2 °C lower than the commercial cotton fabric (Sp3). This phenomenon can be explained by several factors. First, the HPKF experiences greater heat loss due to its unidirectional moisture transfer property, which leads to a gradient in moisture distribution and a high moisture accumulation rate. Second, the acceleration of liquid/sweat evaporation contributes to significant heat dissipation, resulting in a decrease in temperature.

## Conclusions

In summary, we developed an HPKF constructed from sustainable cotton and PLA fibers, featuring an asymmetrical knitting pore design utilizing industrial knitting technology for enhanced intimate comfort. The HPKF not only shows excellent moisture management (OMMC = 0.69) compared to cotton fabric but also achieves a remarkable 27% increase in water evaporation rate. The bilayer structure, with its functional pores in the inner layer, provides outstanding breathability (0.123 KPa·s/m), thermal conductivity, and moisture vapor transmission (10,433 g/m^2^/24 h) that are comparable to cotton. Additionally, the inner layer of the HPKF exhibits excellent antibacterial properties, wherein the inhibition rates against *S. aureus*, *E. coli*, and *C. albicans* can reach up to 99%, 98%, and 93%, respectively. Therefore, this newly developed sustainable HPKF with its asymmetric pore architecture and sustainable fiber material, superior moisture management, and enhanced thermal-wet comfort provides a promising structural platform and functional upgrade for high-performance underwear, potentially replacing conventional cotton-based materials. Future research should focus on quantitative assessments of biodegradability and overall environmental impact, which could also open new opportunities to further enhance the underwear’s sustainability.

## Supplementary Material





## Data Availability

Data will be made available on request.

## Notes

Photograph of the knitting machine; knit chart of porous fabric; specification of cotton and PLA porous fabrics; the weight and thickness of the cotton and PLA porous fabrics; photographs of C0, C10, C5, and C3 and surface morphologies of C0, C10, C5, and C3; photographs of P0, P10, P5, and P3 and surface morphologies of P0, P10, P5, and P3; optical image of a moisture management tester; water location when water is dropped on the inner layer of single-layer fabric C0, C10, C5, and C3; water location when water is dropped on the inner layer of single-layer fabric P0, P10, P5, and P3; water location when water is dropped on the inner C10, C5, C3, and C0 layers of double-layer fabric C0C10, C0C5, C0C3, and C0C0; water location when water is dropped on the inner P10, P5, P3, and P0 layers of double-layer fabric P0P10, P0P5, P0P3, and P0P0; water location when water is dropped on the inner P10, P5, and P3 layers of double-layer fabric C0P10, C0P5, and C0P3; water location when water is dropped on the inner C10, C5, and C3 layers of double-layer fabric P0C10, P0C5, and P0C3; and commercial underwear with different fiber materials (PDF)
Video S1: The manufacturing process of these porous fabrics (MOV)
