# Supplementary material for: Asymmetrical Pore Design of Sustainable Underwear for Improved Intimate Comfort
Source: ACS Omega. 2026 Sep 21;11(39):58908–17. doi: 10.1021/acsomega.6c03941 (PMC13647331; doi:10.1021/acsomega.6c03941)
Supplement: Supplementary file 1 [file ao6c03941_si_001.pdf]

Supporting Information

Asymmetrical Pore Design of Sustainable Underwear for Improved Intimate Comfort

Xiao Tian [a], Ying Li [b], Li Li [b,*]

[a] School of Innovation and Creation Design, Shenzhen Polytechnic University, Shenzhen 518055, China

[b] Division of Integrative Systems and Design, The Hong Kong University of Science and Technology, Hong Kong 999077, China

*Corresponding author: lillyli@ust.hk

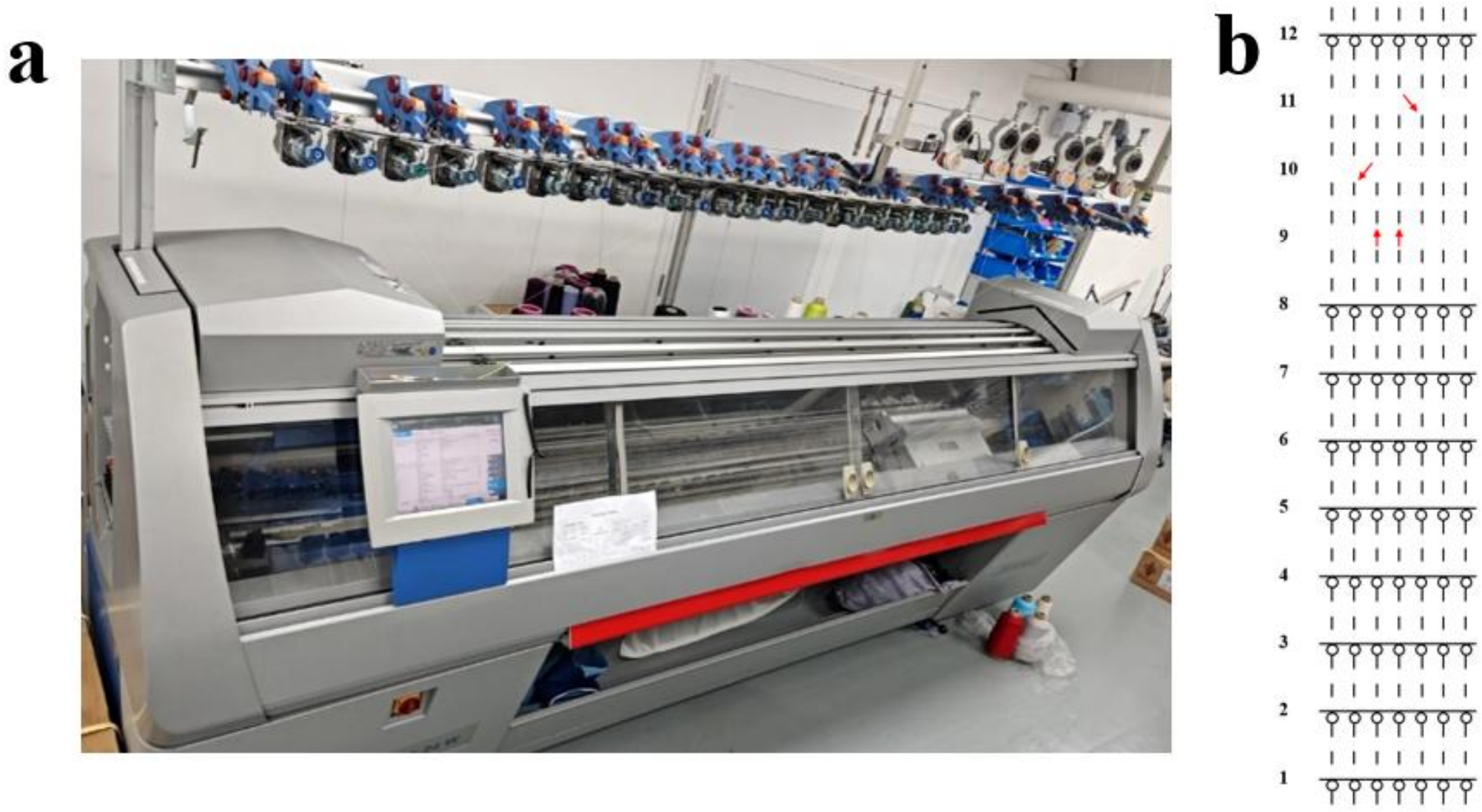

Figure S1. (a) Photo of the knitting machine. (b) Knit chart of porous fabric.

Table S1 Specification of cotton and PLA porous fabrics.

Sample code	Material (Yarn count)	Functional pore distance (mm)	Surface density	
			Wale direction (courses/cm)	Course direction (wales/cm)
C0	Cotton (32s+32s)	-	10	17
C10		10		
C5		5		
C3		3		
P0	PLA (150D)	-	17	17
P10		10		
P5		5		
P3		3		

Note: - means the mentioned fabric has no functional pore.

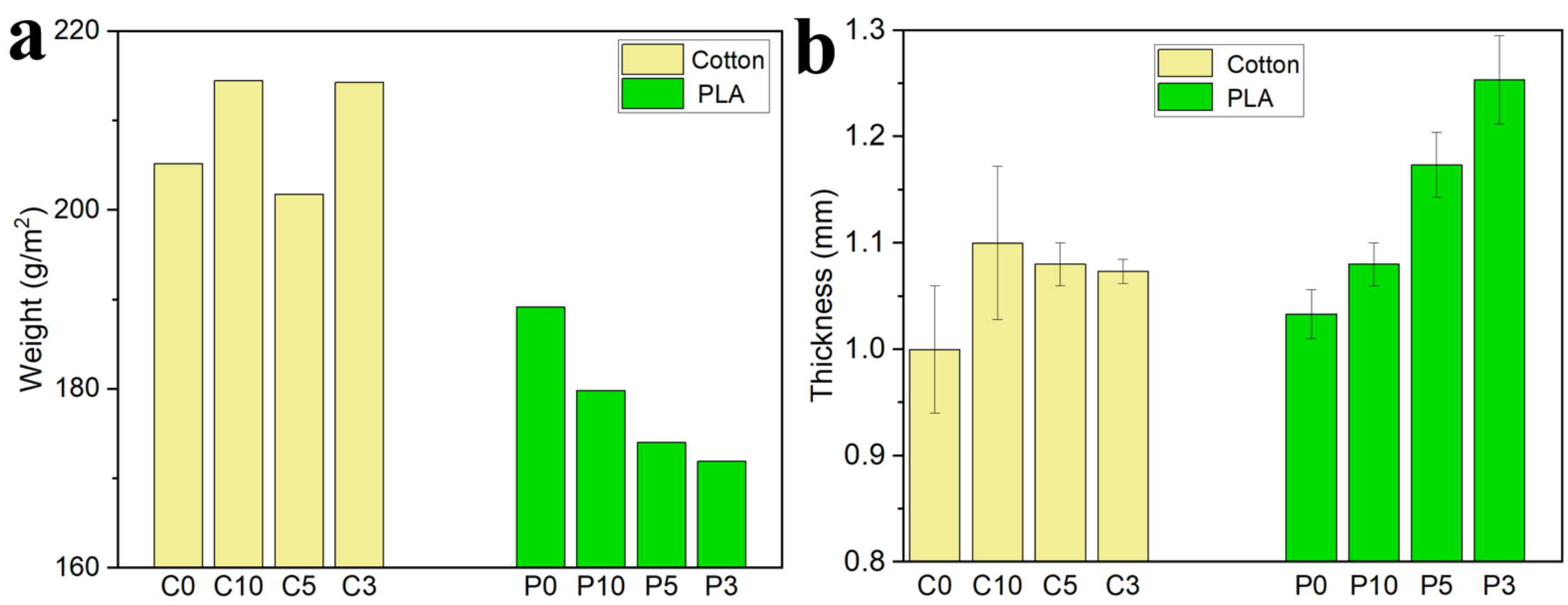

Figure S2. (a) The weight and (b) thickness of the cotton and PLA porous fabrics.

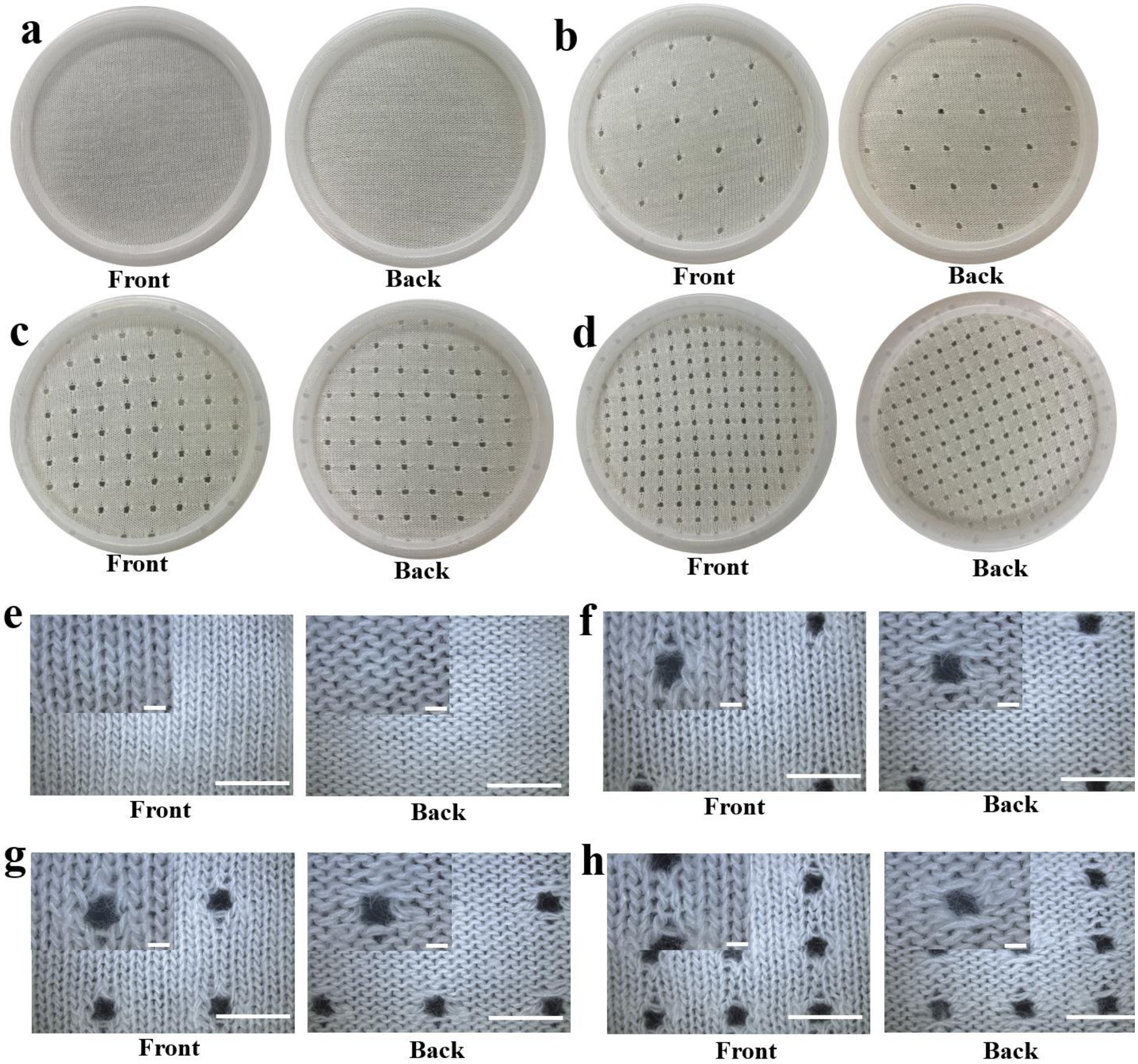

Figure S3. Photos of (a) C0, (b)C10, (c) C5 and (d) C3. Surface morphologies of (e) C0, (f) C10, (g) C5 and (h) C3. Scale bars: 1 mm and 5 mm.

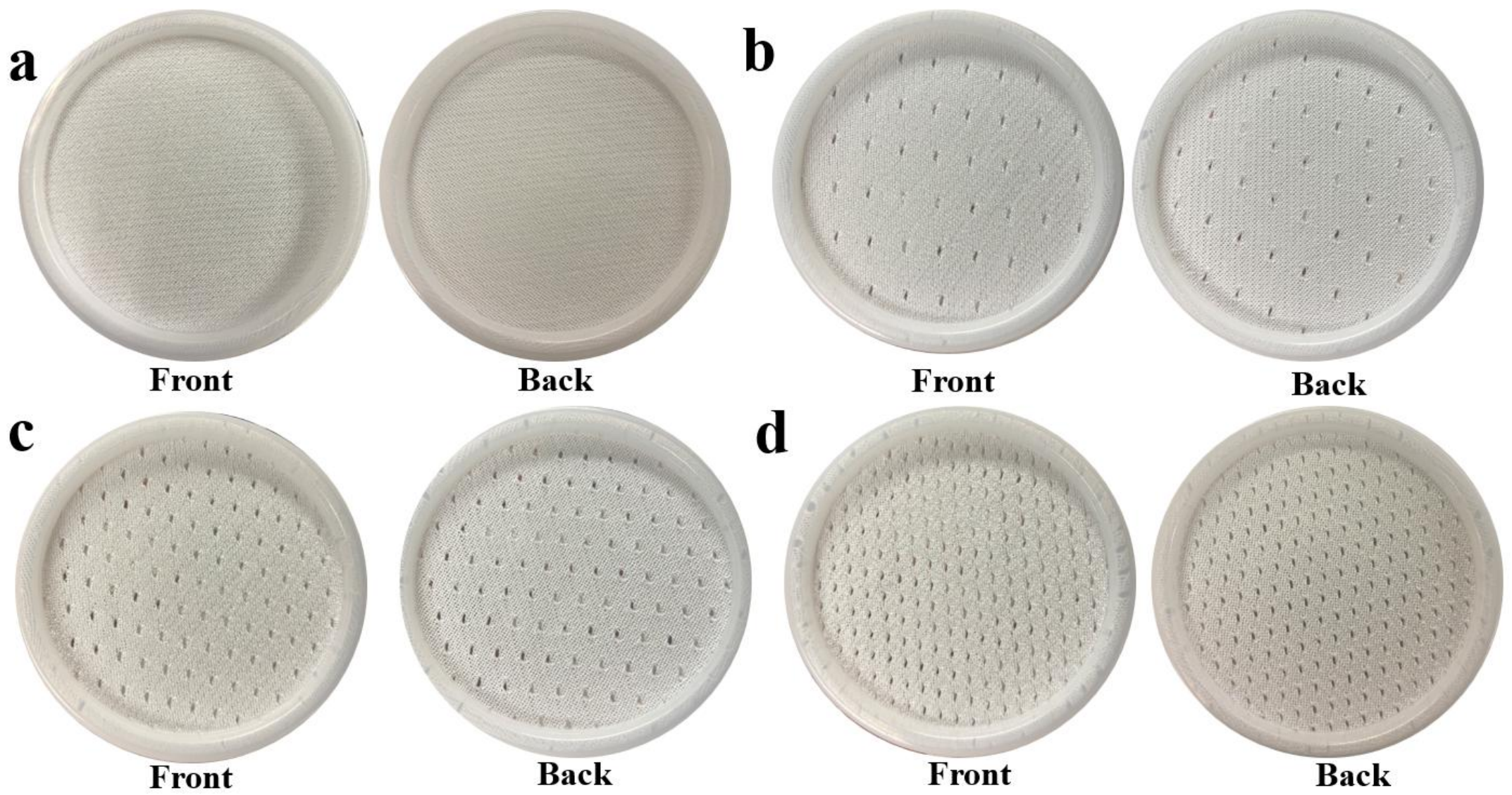

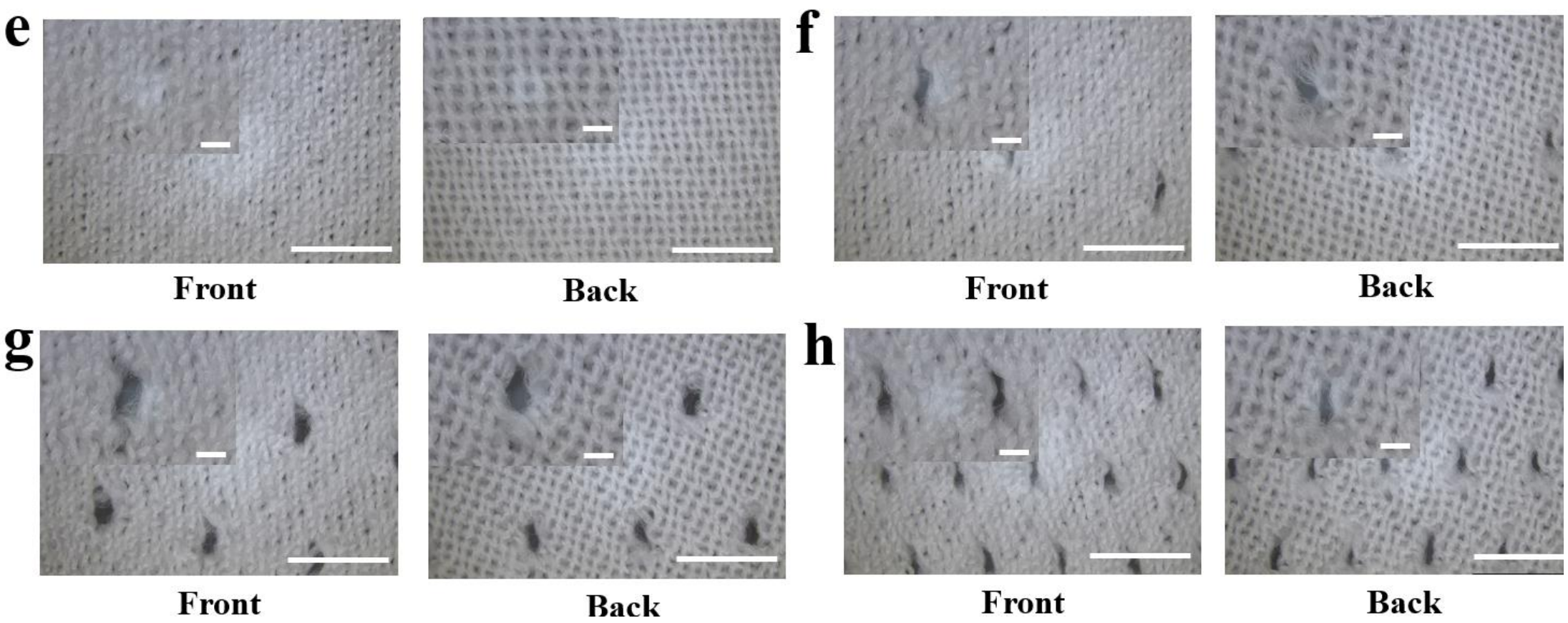

Figure S4. Photos of (a) P0, (b) P10, (c) P5 and (d) P3. Surface morphologies of (e) P0, (f) P10, (g) P5 and (h) P3. Scale bars: 1 mm and 5 mm.

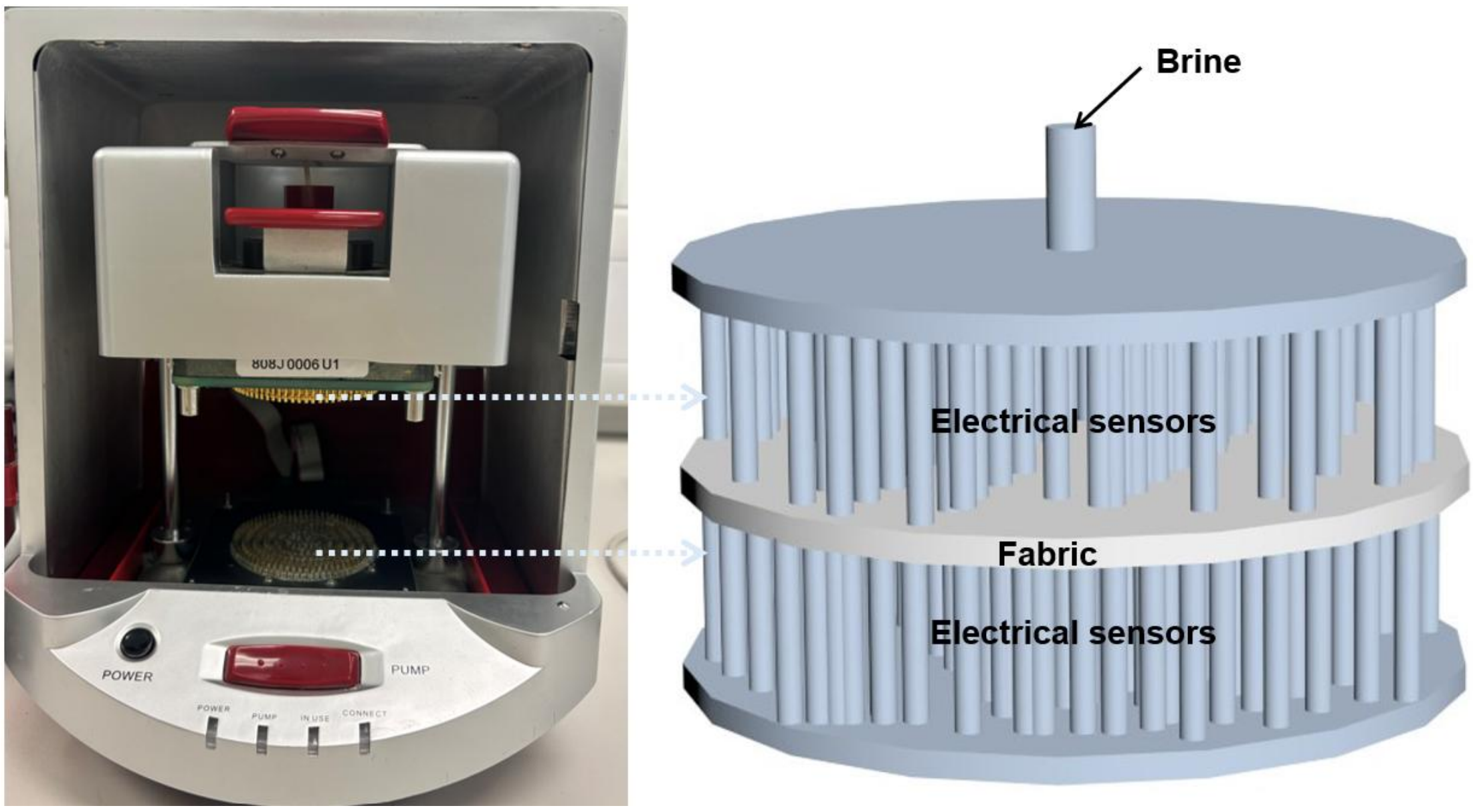

Figure S5. Optical image of a moisture management tester.

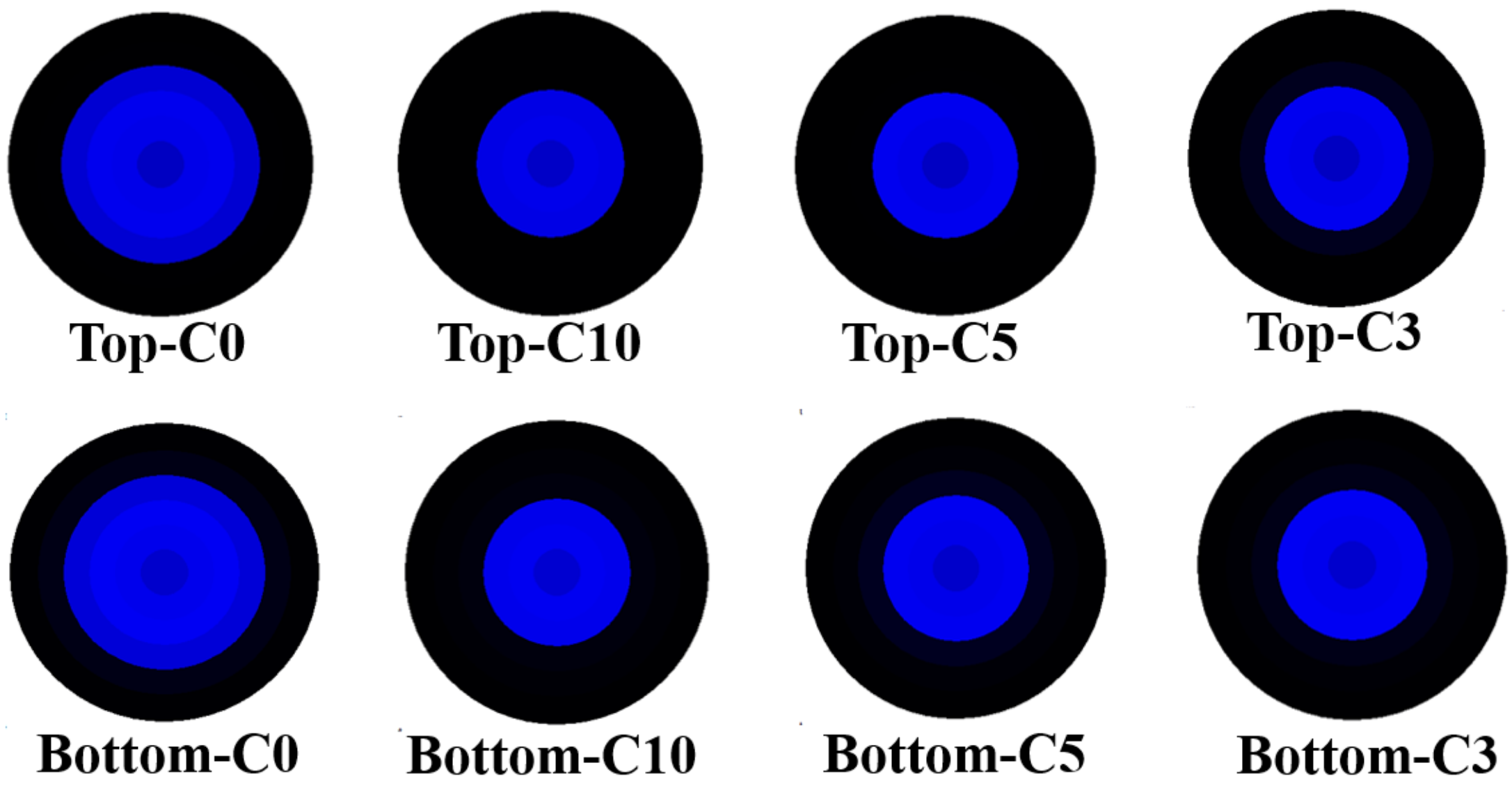

Figure S6. Water location when water is dropped on the inner layer of single layer fabric C0, C10, C5 and C3.

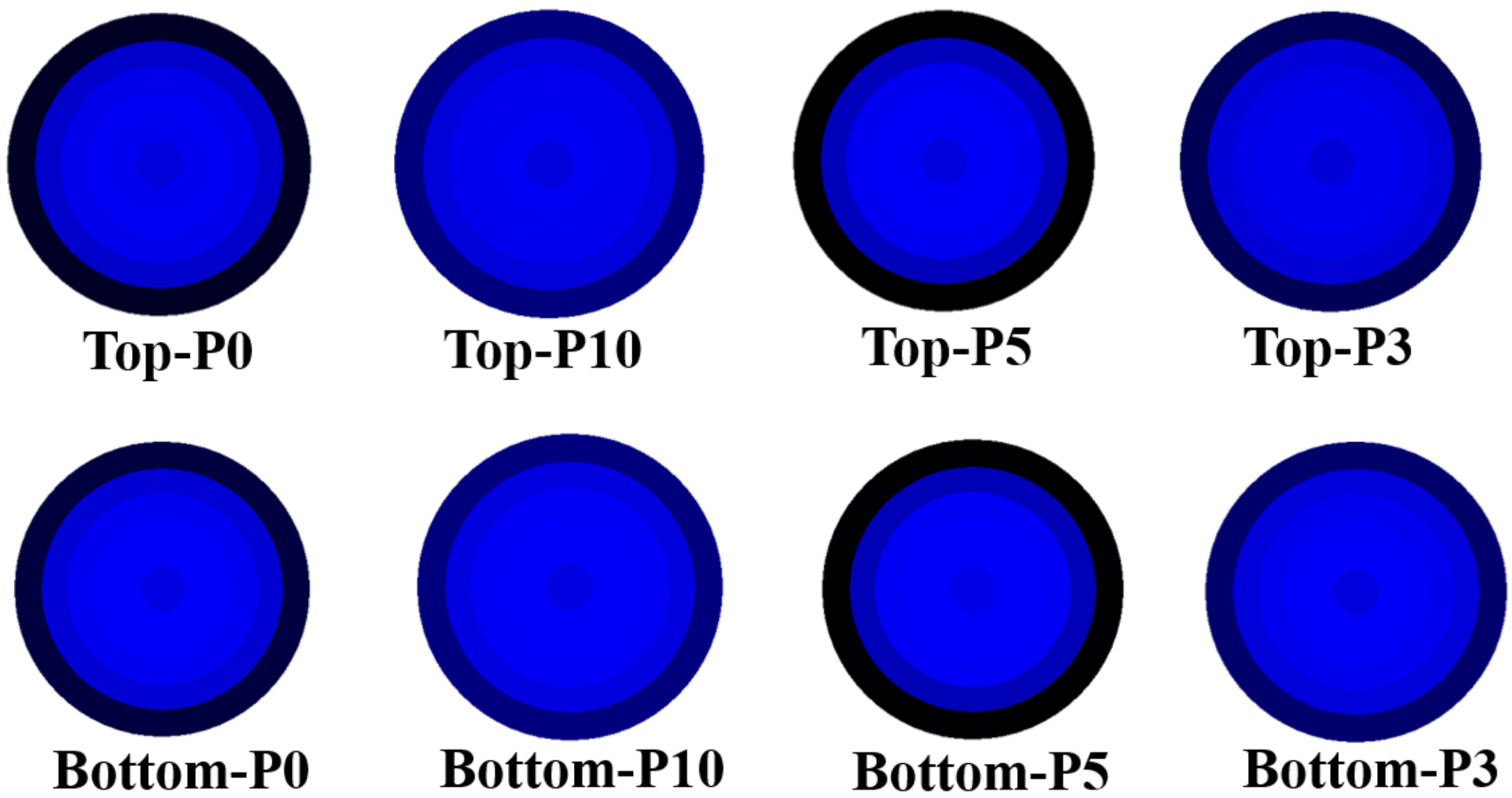

Figure S7. Water location when water is dropped on the inner layer of single layer fabric P0, P10, P5 and P3.

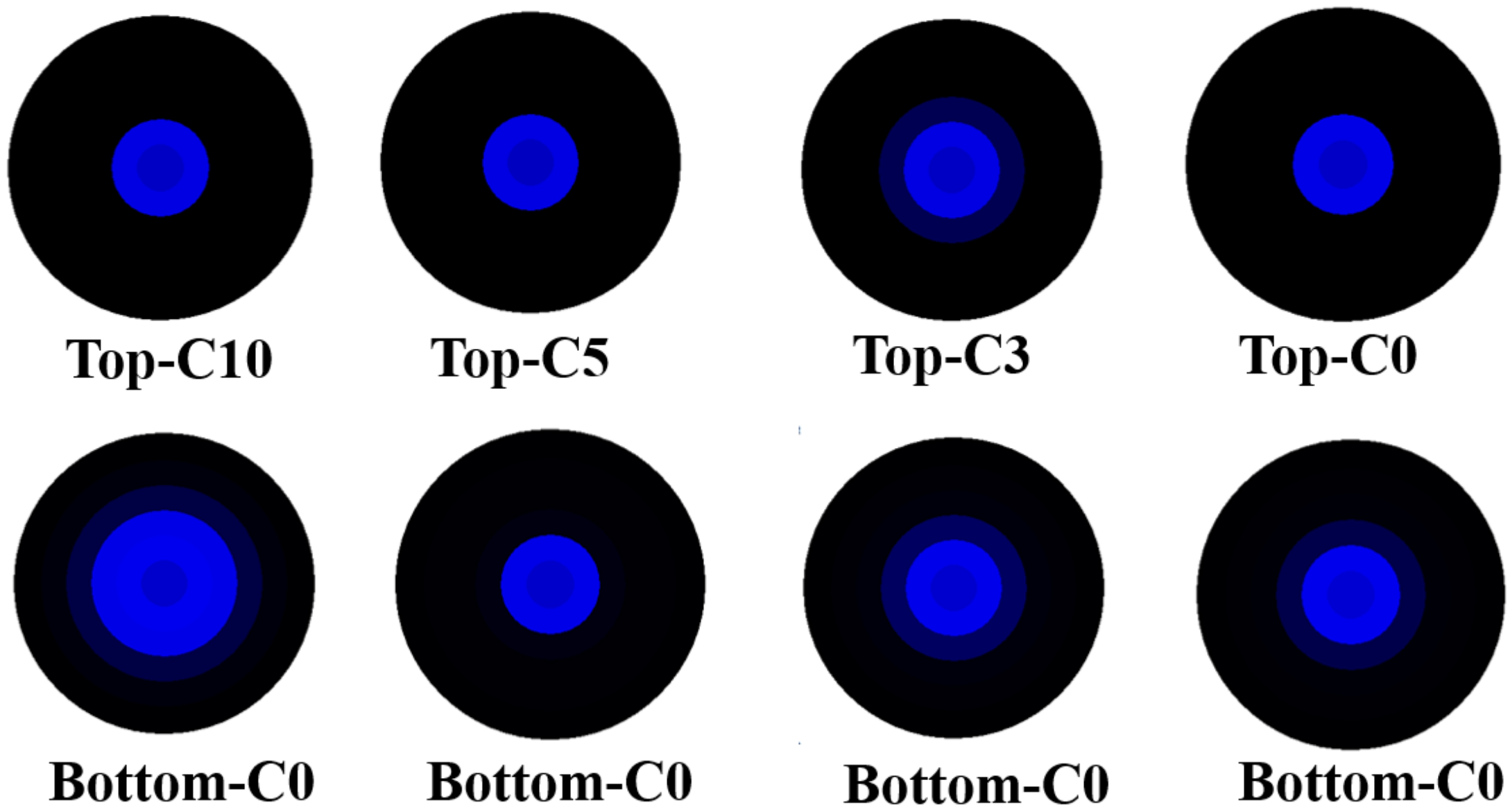

Figure S8. Water location when water is dropped on the inner C10, C5, C3 and C0 layer of double layer fabric C0C10, C0C5, C0C3, and C0C0.

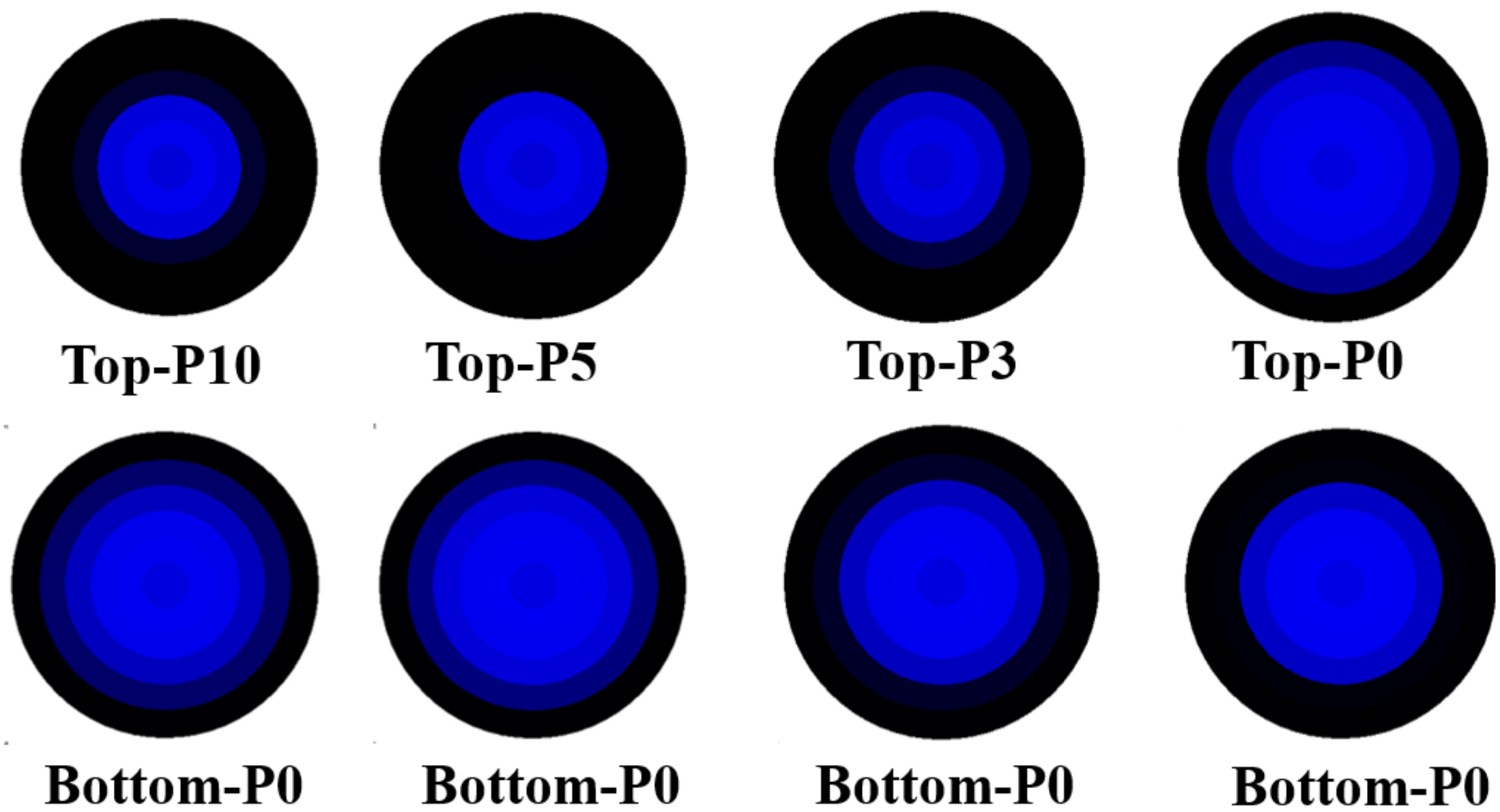

Figure S9. Water location when water is dropped on the inner P10, P5, P3 and P0 layer of double layer fabric P0P10, P0P5, P0P3, and P0P0.

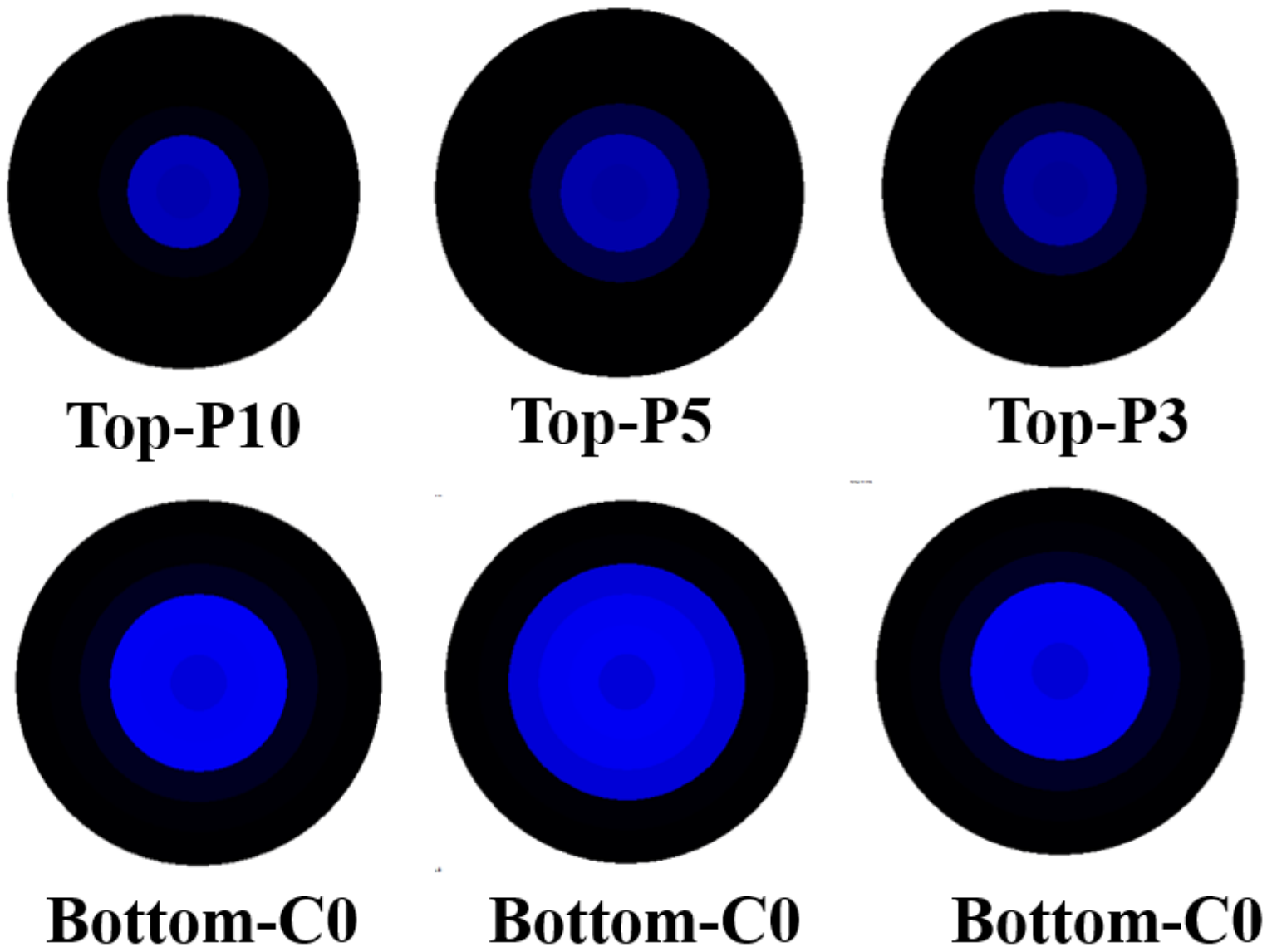

Figure S10. Water location when water is dropped on the inner P10, P5 and P3 layer of double layer fabric C0P10, C0P5, and C0P3.

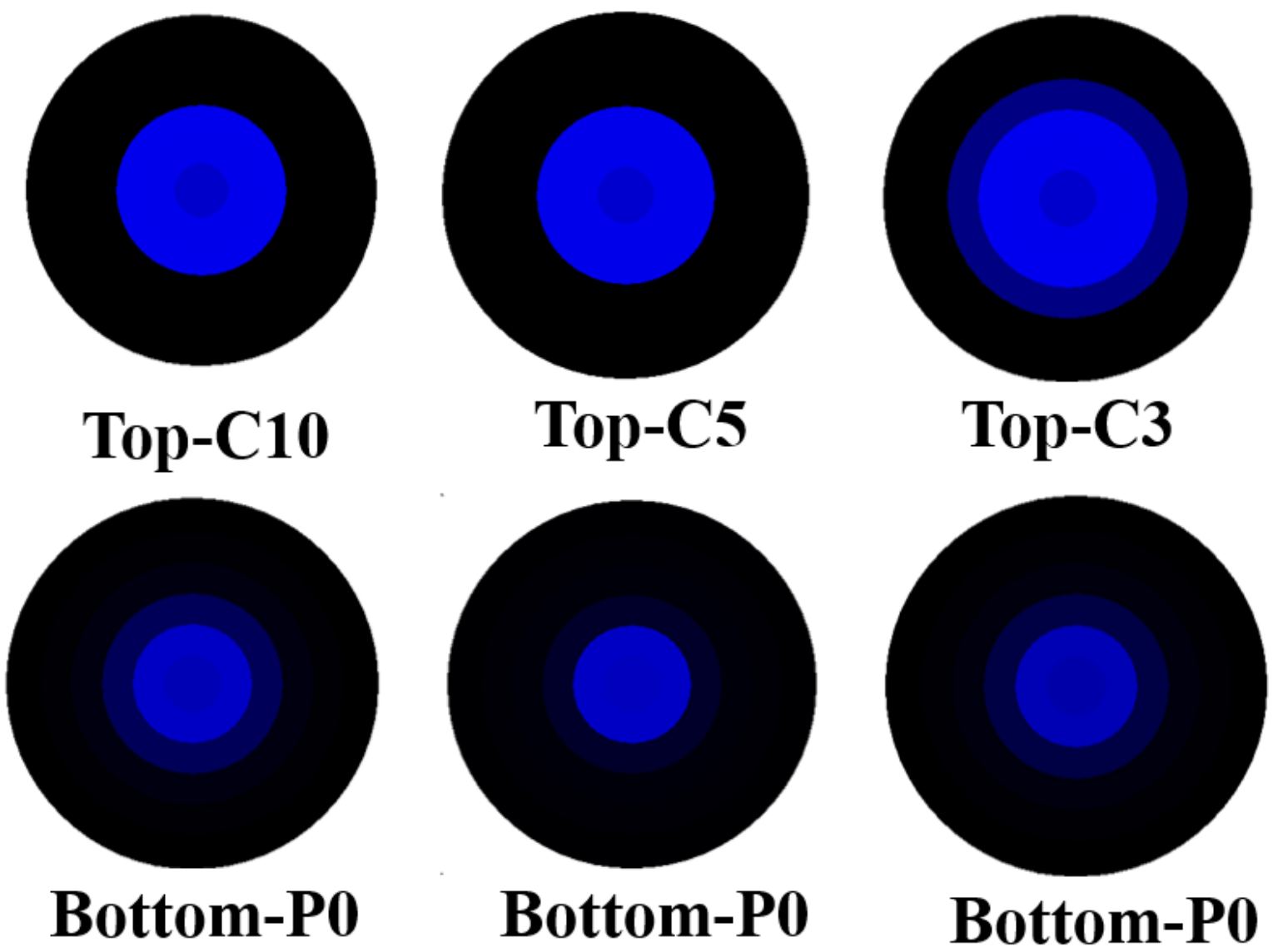

Figure S11. Water location when water is dropped on the inner C10, C5 and C3 layer of double layer fabric P0C10, P0C5, and P0C3.

Table S2 Commercial underwear with different fiber materials.

Sample code	Fiber material	
	Inner layer	Outer layer
1#	100% silk	100% silk
2#	66.4% cotton / 33.6% PLA	93%viscose/7%spandex
3#	88% modal/6%spandex/ 6%silk	95% modal/5%spandex
4#	67%cotton/33% PLA	95% cotton/5%spandex
5#	47.3%viscose/34.3%cotton/15.2%PLA/3.2%silk	51.5% modal/27.7%nylon/20.8%spandex
6#	67.5%Cotton/32.5%PLA	82.2%nylon/17.8%spandex
7#	89.6%viscose/3.9%silk/3.8%spandex/2.7%nylon	95% nylon/5%spandex
8#	100%cotton	95%cotton/5%spandex
9#	65%cotton/31%PLA/4%silk	95%cotton/5%spandex
